# CBL-interacting protein kinase 6 negatively regulates immune response to *Pseudomonas syringae* in Arabidopsis

**DOI:** 10.1093/jxb/erx170

**Published:** 2017-05-24

**Authors:** Atish Sardar, Ashis Kumar Nandi, Debasis Chattopadhyay

**Affiliations:** 1 National Institute of Plant Genome Research, Aruna Asaf Ali Marg, New Delhi, India; 2 School of Life Sciences, Jawaharlal Nehru University, New Delhi, India

**Keywords:** *Arabidopsis thaliana*, CIPK6, ETI, *Pseudomonas syringae*, PTI, salicylic acid

## Abstract

Cytosolic calcium ion (Ca^2+^) is an essential mediator of the plant innate immune response. Here, we report that a calcium-regulated protein kinase Calcineurin B-like protein (CBL)-interacting protein kinase 6 (CIPK6) functions as a negative regulator of immunity against the bacterial pathogen *Pseudomonas syringae* in *Arabidopsis thaliana*. Arabidopsis lines with compromised expression of *CIPK6* exhibited enhanced disease resistance to the bacterial pathogen and to *P. syringae* harboring certain but not all avirulent effectors, while restoration of *CIPK6* expression resulted in abolition of resistance. Plants overexpressing CIPK6 were more susceptible to *P. syringae*. Enhanced resistance in the absence of CIPK6 was accompanied by increased accumulation of salicylic acid and elevated expression of defense marker genes. Salicylic acid accumulation was essential for improved immunity in the absence of CIPK6. CIPK6 negatively regulated the oxidative burst associated with perception of pathogen-associated microbial patterns (PAMPs) and bacterial effectors. Accelerated and enhanced activation of the mitogen-activated protein kinase cascade in response to bacterial and fungal elicitors was observed in the absence of CIPK6. The results of this study suggested that CIPK6 negatively regulates effector-triggered and PAMP-triggered immunity in Arabidopsis.

## Teaser

CBL-interacting protein kinase 6 (CIPK6) negatively regulates the PAMP-triggered and effector-triggered immune response in Arabidopsis.

## Introduction

Plants lack an acquired immune system and rely entirely on the innate immune response. The first line of defense operates by recognizing pathogen/microbe-associated molecular patterns (PAMPs/MAMPs) of the pathogens present at the apoplastic regions using transmembrane pattern recognition receptors (PRRs), and is known as PAMP/MAMP-triggered immunity (PTI/MTI). Well-characterized PRRs in Arabidopsis are Flagellin Sensing 2 (FLS2) and Elongation Factor EF-Tu receptor (EFR) that recognize PAMPs, namely flagellin (flg) and EF-Tu, and activate a common signaling pathway involving activation of the mitogen-activated protein kinase (MAPK) cascade and defense gene transcription (Asai *et al.*, 2002; Boudsocq *et al.*, 2010). Many Gram-negative bacteria inject various effector proteins mostly through the type III secretion system into the host cells to evade PTI. These effectors interfere with the signaling cascades initiated by PAMP recognition to suppress PTI (Alfano and Collmer, 2004; de Torres *et al.*, 2006; He *et al.*, 2006). The other branch, commonly known as effector-triggered immunity (ETI), acts by recognizing the effectors, with the proteins encoded by the plant resistance (R) genes (Belkhadir *et al.*, 2004; Nimchuk *et al.*, 2003; Alfano and Collmer, 2004; Jones and Dangl, 2006; de Torres *et al.*, 2006; He *et al.*, 2006). Pathogen effectors that activate the R proteins, and thereby the immune response, are called avirulent (Avr) proteins. Some of these R proteins have a central NB-LRR (nucleotide binding-leucine-rich repeat) domain with the N-termini having homology to Toll and interleukin receptors (TIR-NB-LRR), or have a coiled-coil motif (CC-NB-LRR) (Ting *et al.*, 2008; Eitas and Dangl, 2010). Activation of R proteins results in induction of a strong immune response culminating in local and systemic changes in gene expression, increased salicylic acid (SA) level, NADPH-oxidase-dependent oxidative burst, and sometimes cell death, known as the hypersensitive response (HR) (Grant *et al.*, 2000; Jones and Dangl, 2006; Torres *et al.*, 2006; Hein *et al.*, 2009).

The ubiquitous plant pathogen *Pseudomonas syringae* pv. *tomato* DC3000 (*Pst* DC3000), widely used as a surrogate for studying mechanism of various effector functions, secretes various effectors, including AvrPto and AvrPtoB, through the type III secretion system (Shan *et al.*, 2008; Cheng *et al.*, 2011). In a resistant host such as a resistant tomato variety, AvrPto and AvrPtoB are recognized by Pto, a serine-threonine kinase. Pto kinase is required for activation of Prf, an NB-LRR protein, leading to cell death and disease resistance (Mucyn *et al.*, 2006; Eitas and Dangl, 2010; Oh and Martin, 2011). Enhanced disease susceptibility 1 (EDS1), a lipase like protein, is indispensable for immunity mediated by R proteins having TIR-NB-LRR domains such as RPS4. A type III effector AvrRps4 (from *P. syringae* pv. *phaseolicola*) disrupts the protein complex made by EDS1 and RPS4 by interacting with EDS1 (Bhattacharjee *et al.*, 2011; Heidrich *et al.*, 2011). Two type III effectors AvrRpm1 (from *P. syringae* pv. *maculicola*) and AvrB (from *P. syringae* pv. *glycinea*) target a host plasma membrane-associated protein RIN4 and induce phosphorylation of RIN4. This RIN4 modification activates a CC-NB-LRR protein RPM1 (Belkhadir *et al.*, 2004). Similarly, another effector protein AvrRpt2 (from *P. syringae* pv. *tomato* JL1065), a cysteine protease, cleaves RIN4 and the cleaved products are sensed by another CC-NB-LRR protein RPS2. RIN4-mediated defense signaling requires NDR1, a RIN4-interacting membrane protein (Mackey *et al.*, 2002; Axtell *et al.*, 2003; Chisholm *et al.*, 2005; Kim *et al.*, 2005; Day *et al.*, 2006). Signaling cascades mediated by R proteins of both CC-NB-LRR and TIR-NB-LRR types converge at EDS5, a MATE-family protein, ultimately causing elevation of SA accumulation. EDS5 expression in response to pathogen infection is dependent on EDS1 and NDR1, and its expression is essential for pathogen-mediated induction of the SA level in the host cell (Nawrath *et al.*, 2002). Transcript of isochorismate synthase 1 (*ICS1*), a major SA biosynthetic gene, is quickly accumulated upon infection. Lesions in the *ICS1* gene (*sid2-1* and *sid2-2*) resulted in impairment of pathogen-induced SA accumulation (Nawrath and Métraux, 1999; Wildermuth *et al.*, 2001).

A rapid and sustained increase in the cytosolic calcium level [Ca^2+^]_cyt_ is necessary for pathogen response (Grant *et al.*, 2000; Lecourieux *et al.*, 2006). Direct regulation of the SA level by a Ca^2+^-binding protein has been demonstrated using AtSR1, which negatively regulated the pathogen-induced SA level (Du *et al.*, 2009). The Calcineurin B-like protein (CBL) family is a group of Ca^2+^ sensors which is activated by Ca^2+^ to initiate various signaling processes. Recently, tomato CBL10 and its interacting partner *Sl*CIPK6 were shown to function as a positive regulator of Pto/AvrPto-mediated programmed cell death in tomato through activation of Respiratory burst oxidase homolog B (RbohB) upon infection with *Pst* DC3000 (de la Torre *et al.*, 2013). In contrast to tomato, AvrPto does not trigger ETI in Arabidopsis [Columbia-0 (Col-0)], which is a susceptible host to this pathogen (Hauck *et al.* 2003). In this study, we report that CIPK6 (AT4G30960), a Ca^2+^-regulated protein kinase functions as a negative regulator of PTI and the SA-mediated immune response against *Pst* DC3000 in Arabidopsis.

## Materials and methods

### Plant materials and growth conditions

*Arabidopsis thaliana* T-DNA insertion lines *cipk6kd* (SALK_08095) and *cipk6* (GK-448C12-CS442948) have been described earlier (Tripathi *et al.*, 2009; Held *et al.*, 2011). Loss-of-function mutants *sid2-1* (Wildermuth *et al.*, 2001) and *eds1-2* (Parker *et al.*, 1996) were procured from Dr V. Bonardi, University of North Carolina, Chapel Hill, USA and Dr Saikat Bhattacharjee, Regional Centre for Biotechnology, Faridabad, India. The *CIPK6* gene (*CIPK6* lacks an intron) without or with the 2.1 kb long promoter region was amplified by PCR to construct *35S::CIPK6* or *PCIPK6::CIPK6* in pCAMBIA1305.1 with or without the *Cauliflower mosaic virus* (CaMV) 35S promoter, respectively. The *Agrobacterium* strain GV3101 harboring *CIPK6* constructs was used to transform the wild-type (Col-0) and *cipk6* plants by floral dip infiltration as described previously (Clough and Bent, 1998). T_3_/T_4_ single-insertion homozygous lines were used. The presence of the transgene and its expression were confirmed by PCR and reverse transcription–PCR (RT–PCR), respectively. Seeds of the wild type (Col-0), RNAi, T-DNA insertion, mutant, and overexpressing Arabidopsis lines were stratified for 2 d at 4 °C, sown on soil, and grown in controlled-environment chambers (Conviron, Winnipeg, Canada) set to 22–24 °C, 70% humidity with a 10 h light/14 h dark photoperiod (100 µmol µm^−2^ s^−1^ light) for 4–5 weeks.

### *Generation of the* CIPK6 *RNAi construct*

To generate the *CIPK6* hairpin RNAi transformation construct, a 409 bp fragment of the second intron of the *BjMYB28-3* gene was amplified using specific primers having *Xba*I/*Hin*dIII sites and were cloned into pGEMT-easy vector (Augustine *et al.*, 2013). To this construct, a 334 kb fragment covering the ORF and 3'-untranslated region (UTR) of *CIPK6* (encompassing base pairs 1160–1493 of the *CIPK6* gene) was amplified and cloned in both sense and antisense orientations to create an RNAi cassette. This cassette was excised and cloned directionally at *Nco*I/*Spe*I sites of the binary vector pCAMBIA1305.1 to develop the RNAi construct. The transformation and subsequent selection methods were followed as described previously. All the primers used in this study are listed in Supplementary Table S1 at *JXB* online.

### Bacterial infection

The bacterial pathogen *Pseudomonas syringae* pv. *tomato* DC3000 (*Pst* DC3000) and its type III secretion-deficient mutant, Δ*hrcC*, were provided by Professor G.B. Martin, Boyce Thompson Research Institute, Ithaca, NY, USA. The bacteria carrying an empty vector (EV) or constructs encoding various type III effectors (*AvrB*, *AvrRpt2*, *AvrRpm1*, and *AvrRps4*) were grown on King’s medium B agar plates or in liquid medium supplemented with 50 µg ml^–1^ rifampicin and 50 µg ml^–1^ kanamycin at 28 °C. The bacterial culture was resuspended in 10 mM MgCl_2_ and manually infiltrated in leaves with an OD_600_ of 0.0005 for the virulent strain, *Pst* DC3000 (EV) and an OD_600_ of 0.001 for other strains. Crushed leaf samples were serially diluted with 10 mM MgCl_2_ and plated onto King’s B medium containing the appropriate antibiotics for bacterial counts. The values presented are the mean of at least three biological replicates. At least eight representative leaves for each plant type and five technical replicates were used to generate results. Statistical analyses were performed using two-way ANOVA (Fujikoshi, 1993).

### Gene expression analysis

Gene-specific primers were used to detect transcripts by qRT–PCR. All primers were designed using PRIMER EXPRESS version 3.0 (Applied Biosystems, Foster City, CA, USA) with default parameters. The primers used are listed in Supplementary Table S1. qRT–PCRs were performed with 2× SYBR Power Green master mix using the ViiA 7 system (Applied Biosystems) according to the manufacturer’s instructions. The specificity of amplicons was verified by melting curve analysis. At least three biological replicates and three technical replicates for each sample were used. *ACTIN 2* (At3g18780) and *TUBULIN 4* (At5g44340) were used as the reference gene internal controls. Relative expression was calculated according to the ΔΔCt method.

### Reactive oxygen species (ROS) detection and measurement

Production of hydrogen peroxide was visualized *in situ* by 3,3'-diaminobenzidine (Sigma-Aldrich) staining. Leaves of 4- to 5-week-old Arabidopsis plants were manually infiltrated with *Pst* DC3000 (EV) and related bacterial strains (OD_600_=0.02), and the plants were incubated for 5 h before DAB staining. Five to six leaves were vacuum-infiltrated with a solution containing 1 mg ml^–1^ DAB and incubated for 4 h. Leaves were then de-stained in a solution of 3:1:1 ethanol/lactic acid/glycerol for visualization. Leaf samples for each plant type were constituted of three leaves per plant from four independent plants. To perform ROS burst kinetics, the third, fourth, and fifth true leaves of 4 week-old Arabidopsis plants were sampled with a cork borer (1.1 cm^2^) and floated adaxial side up overnight on sterile water. Prior to elicitation, bacteria were scraped from plates and washed twice in sterile distilled water, and the final concentration of bacterial elicitation solution was adjusted to 1 × 10^8^. Water was replaced with 100 µl of the elicitation solution containing 0.2 μM luminol (Sigma-Aldrich), 20 μg ml^–1^ horseradish peroxidase (HRP; Sigma-Aldrich), and the appropriate bacterial strain. *Pst*-induced ROS production was measured *in vivo* as luminescence using a POLARstar Omega (BMG Labtech, UK) 96-well microplate luminometer every 42 s up to 250 min. The values presented are the mean of six biological replicates.

### Quantification of salicylic acid

Free SA and glucose-conjugated SA (SAG) measurements were performed using a biosensor system as described before (Defraia *et al.*, 2008). In brief, leaves (100 mg) were collected at 24 hours post-infiltration (hpi; for avirulent effectors) or 9 hpi [*Pst* DC3000 and Pst DC3000 (Δ*hrcC*)] and frozen in liquid nitrogen. The tissue was homogenized in 200 μl of 0.1 M acetate buffer (pH 5.6). One aliquot (100 μl) of the supernatant was used for free SA measurements, and another was incubated with 4 U of β-glucosidase (Sigma-Aldrich) for 90 min at 37 °C for total SA measurement. A 20 μl aliquot of each plant extract and standard SA solutions (prepared in *sid2-1* extract) were added to the assigned wells of a black cell culture plate. A 50 μl aliquot of *Acinetobacter* sp. ADPWH_*lux* (OD_600_ of 0.4) was added to each well, incubated at 37 °C for 1 h, and luminescence readings for each sample was taken using POLARStar Omega (BMG Labtech). Leaf samples for each plant type constitute three leaves per plant from six independent plants, and the values presented are the mean of three biological replicates.

### PAMP treatment

Leaves of 4- to 5-week-old Arabidopsis plants were manually infiltrated with flg22 (1 µM) or water. Leaf samples were then taken at 0, 5, and 15 min time points for both Col-0 and *atcipk6*. Tissue samples were ground in liquid nitrogen and resuspended in 50 mM Tris–HCl (pH 7.5), 5 mM EDTA, 5 mM EGTA, 150 mM NaCl, 1 mM DTT, 10 mM Na_3_VO_4_, 10 mM NaF, 50 mM β-glycerolphosphate, 1 mM phenylmethylsulfonyl fluoride (PMSF), plant protease inhibitor cocktail (Sigma-Aldrich), 10% glycerol, 0.1% NP-40. After centrifugation, the protein supernatant was mixed with Laemmli sample buffer and boiled for 5 min. Active forms of MAPK3 and MAPK6 were then detected by western blotting using pTEpY antibody (Cell Signaling Technology, #A9101). Total MPK3 and MPK6 proteins were detected by western blotting using anti-AtMPK3 and anti-AtMPK6 (Sigma-Aldrich, #M8318, #A7104) as primary antibodies, respectively, and goat anti-rabbit–HRP (Amersham Biosciences) as secondary antibody. For gene expression analysis, seedlings were grown on a plate for 8 d and then transferred to liquid Murashige and Skoog (MS) medium (0.5× MS, 0.25% sucrose, and 1 mM MES pH 5.7) in 12-well plates for equilibration for 48 h to allow recovery from stress caused during the transfer, and then treated with flg22 (1 µM). Seedlings were treated similarly for other PAMPs, elf18 (1 µM), chitin (50 µg ml^–1^), or peptidoglycan (PGN; 50 µg ml^–1^). For MAPK activity assay, leaves of 4-week-old plants were manually infiltrated with 1 µM flg22.

## Results

### *CIPK6 negatively regulates resistance to* Pst *DC3000 in Arabidopsis*

**Fig. 1.**
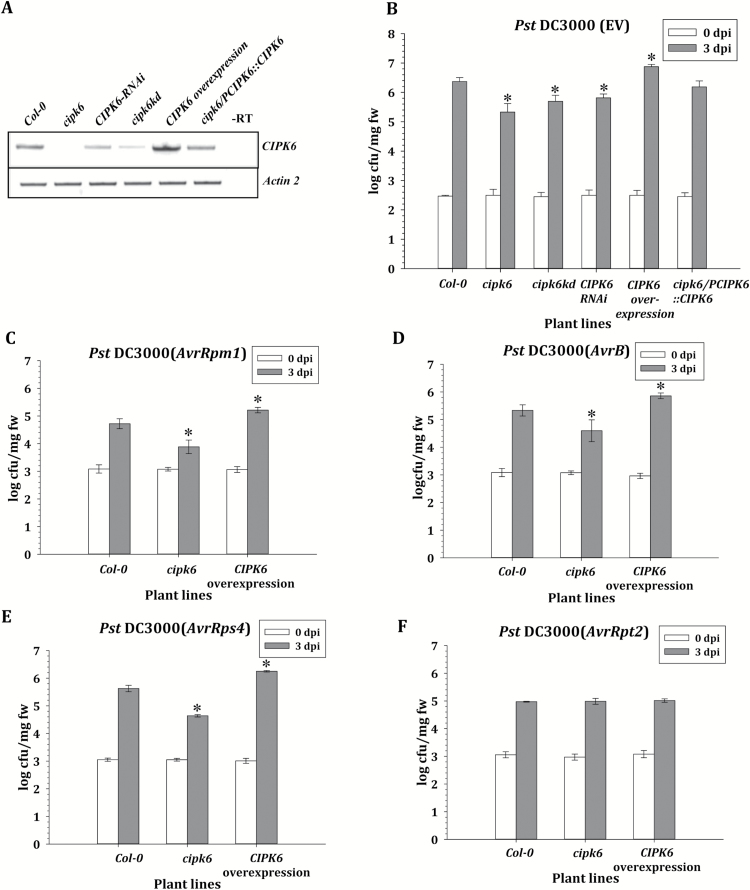
CIPK6 is a negative regulator of plant defense. (A) Expression of *CIPK6* in the wild type (Col-0) and different Arabidopsis lines used in this study as determined by RT–PCR. A lane with RNA from Col-0 without using reverse transcriptase (–RT) is shown to rule out genomic DNA contamination. *Actin2* transcript was used as the control. (B) Bacterial growth content assay. *Pst* DC3000 (empty plasmid vector, EV) (OD_600_=0.0005) was manually infiltrated into leaves of the various Arabidopsis plants mentioned. Bacterial growth was assessed at 0 and 3 days post-infection (dpi) and was expressed as log of colony-forming unit per miligram of fresh weight (log cfu/mg fw). The asterisks indicate a significant difference following two-way ANOVA (α=0.05). (C–F) *Pst* DC3000 expressing *AvrRpm1*, *AvrB*, *AvrRps4*, or *AvrRpt2* from plasmid vectors were manually infiltrated (OD_600_=0.001) into the rosette leaves of Col-0, *cipk6*, and *CIPK6OX* plant lines. Bacterial growth was assessed at 0 and 3 dpi and is presented similarly to as described above.

Pto, a tomato ser/thr kinase, recognizes *Pst* DC3000 effectors AvrPto and AvrPtoB, and elicits AvrPto/Pto-triggered immunity in resistant tomato varieties (Mucyn *et al.*, 2006; Oh and Martin, 2011). In contrast, delivery of AvrPto does not show AvrPto/Pto-mediated immunity, but rather suppresses the defense response and promotes bacterial growth in Arabidopsis (Hauck *et al.*, 2003; de Torres *et al.*, 2006). Tomato CIPK6 was shown to regulate AvrPto/Pto-triggered immunity positively (de la Torre *et al.*, 2013). To investigate the role of CIPK6 in the immune response of Arabidopsis, leaves of two Arabidopsis T-DNA insertion lines, a *CIPK6* RNAi line, and a CIPK6-overexpressing line (*CIPK6OX*), which do not express (*cipk6*), partially express (*cipk6kd*, *CIPK6-RNAi*), or highly express *CIPK6* (Fig. 1A), were infiltrated with *Pst* DC3000. Almost 10- and 5-fold lower bacterial counts were observed in *cipk6* and *cipk6kd* lines, respectively, in comparison with that in the wild-type (Col-0) plants at 3 days post-infiltration (dpi), while the RNAi line also showed a similar bacterial count to the *cipk6kd* line (Fig. 1B). Lower bacterial growth in the Arabidopsis lines with no or low *CIPK6* expression was also evident from less chlorosis in the infected leaves (Supplementary Fig. S1). Leaves of *cipk6* plants transformed with the *CIPK6* cDNA under the control of the native promoter (*PCIPK6*::*CIPK6*) did not show any significant difference in bacterial titer and chlorosis with respect to the wild-type (Col-0) plants. In contrast, an ~5-fold higher bacterial count and severe chlorosis were observed in the wild-type plants expressing *CIPK6* under the CaMV 35S promoter (*CIPK6OX*) (Fig. 1B; Supplementary Fig. S1). Various other bacterial effectors such as AvrRps4, AvrRpt2, AvrRpm1, and AvrB induce ETI in Arabidopsis (Jones and Dangl, 2006; Xin and He, 2013). Therefore, *Pst* DC3000 strains carrying these individual effectors were infiltrated in the leaves of Col-0, *cipk6*, and *CIPK6OX* plants. The *cipk6* line showed 6- to 10-fold reduced bacterial growth for *Pst* DC3000-*AvrRps4*, -*AvrRpm1*, and -*AvrB* as compared with the wild-type plants, while *CIPK6OX* plants displayed an ~5-fold higher bacterial count at 3 dpi. No significant difference in bacterial titer was observed for *AvrRpt2* between the wild type and *cipk6* line, suggesting that Arabidopsis CIPK6 is not a general negative regulator of plant defense, but rather functions differentially in distinct effector-driven signaling pathways (Fig. 1C–F).

### *CIPK6 negatively regulates expression of the pathogenesis-related gene* PR1

**Fig. 2.**
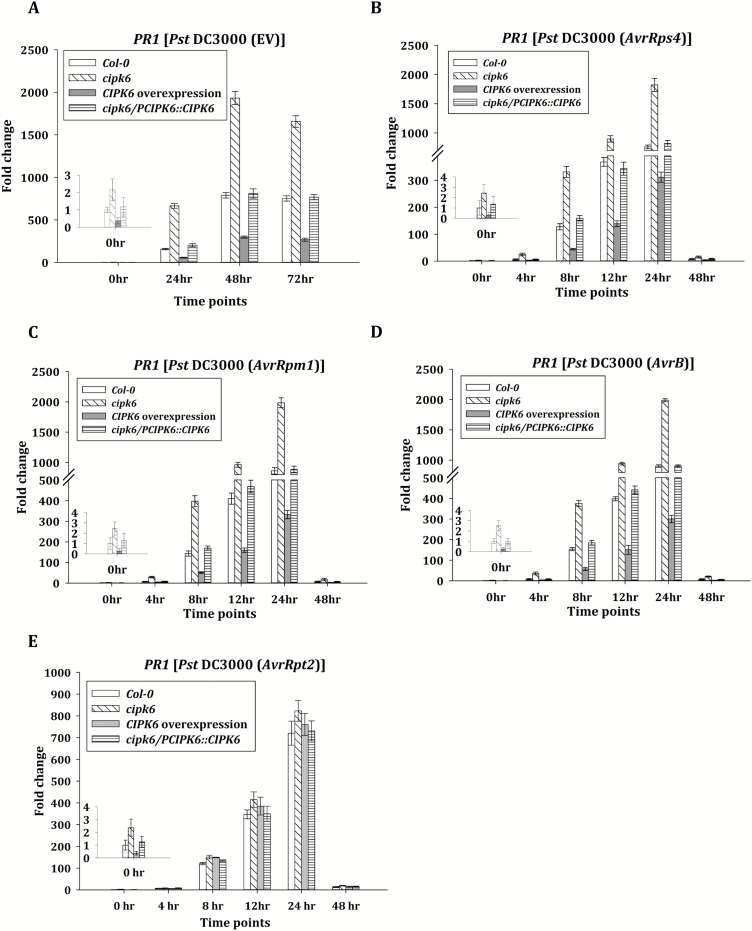
Expression of the defense marker gene *PR1* was enhanced in the absence of CIPK6. (A–E) Time course of *PR1* expression assessed by qRT–PCR in Col-0, *cipk6*, *CIPK6* overexpression, and *cipk6/PCIPK6::CIPK6* plant lines in response to *Pst* DC3000 (EV) and to *Pst* DC3000 (*AvrRps4*/*AvrRpm1*/*AvrB*/*AvrRpt2*) infiltration. *Actin 2* and *Tubulin 4* were used as internal controls.

The *CIPK6* gene expression level in response to *Pst* DC3000 infiltration decreased with time and was decreased by 3-fold at 12 hpi (Supplementary Fig. S2). This is in contrast to the *CIPK6* expression pattern in tomato, in which the *SlCIPK6* expression level rapidly increased by 4-fold within 4 h of infection (de la Torre *et al.*, 2013). The *CIPK6* expression pattern has been reported to vary in different plants, indicating its diverse functions in different species (Kim *et al.*, 2007; Quan *et al.*, 2007; Tripathi *et al.*, 2009*a*). Resistance against bacterial pathogen is accompanied by the expression of the *PR1* gene, a marker for activation of SA signaling (Cameron *et al.*, 1999). In agreement with enhanced resistance, *cipk6* mutant plants showed a markedly higher level of *PR1* expression, while *CIPK6OX* plants exhibited a >3-fold lower expression level compared with wild-type and *cipk6/PCIPK6::CIPK6* plants (Fig. 2A) Similar differential *PR1* expression was observed in Col-0, *cipk6*, *CIPK6OX*, and *cipk6/PCIPK6::CIPK6* plants in response to *Pst* DC3000-*AvrRps4*, -*AvrB*, and -*AvrRpm1* infection, suggesting that CIPK6 negatively regulates effector-triggered defense signaling in Arabidopsis (Fig. 2B–D). In agreement with the bacterial titer described before, lack of expression or high expression of *CIPK6* did not affect *PR1* expression in the case of *Pst* DC3000-*AvrRpt2* infection (Fig. 2E).

### *Resistance in* cipk6 *plants is dependent on salicylic acid accumulation*

**Fig. 3.**
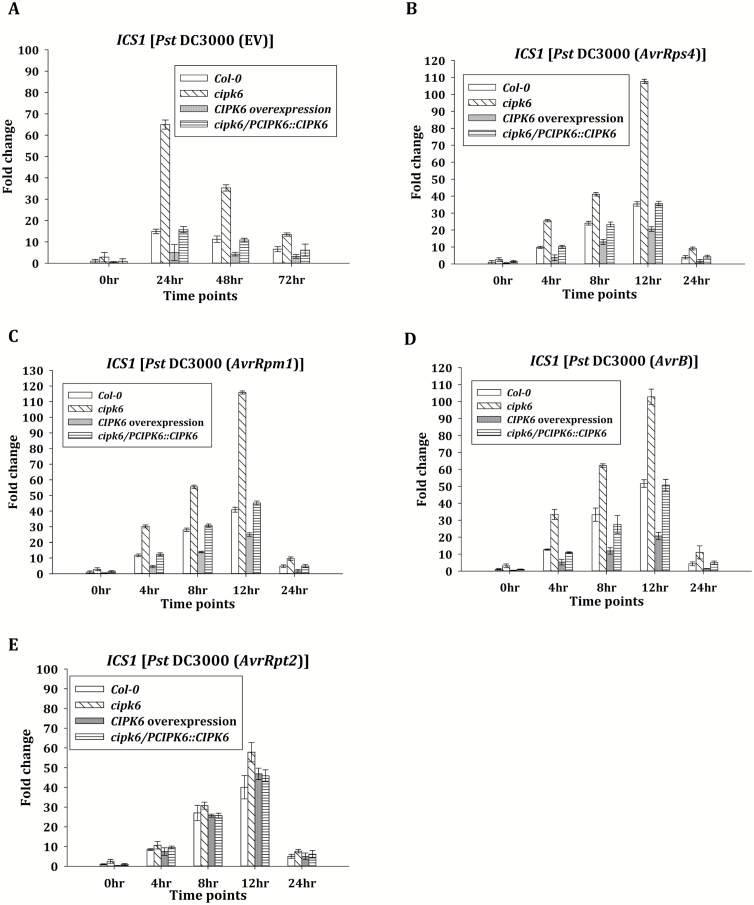
Expression of the SA biosynthetic gene *ICS1* was enhanced in the absence of CIPK6. (A–E) Time course of *ICS1* expression assessed by qRT–PCR in Col-0, *cipk6*, *CIPK6* overexpression, and *cipk6/PCIPK6::CIPK6* plant lines in response to *Pst* DC3000 (EV) and to *Pst* DC3000 (*AvrRps4*/*AvrRpm1*/*AvrB*/*AvrRpt2*) infiltration. *Actin 2* and *Tubulin 4* were used as internal controls.

**Fig. 4.**
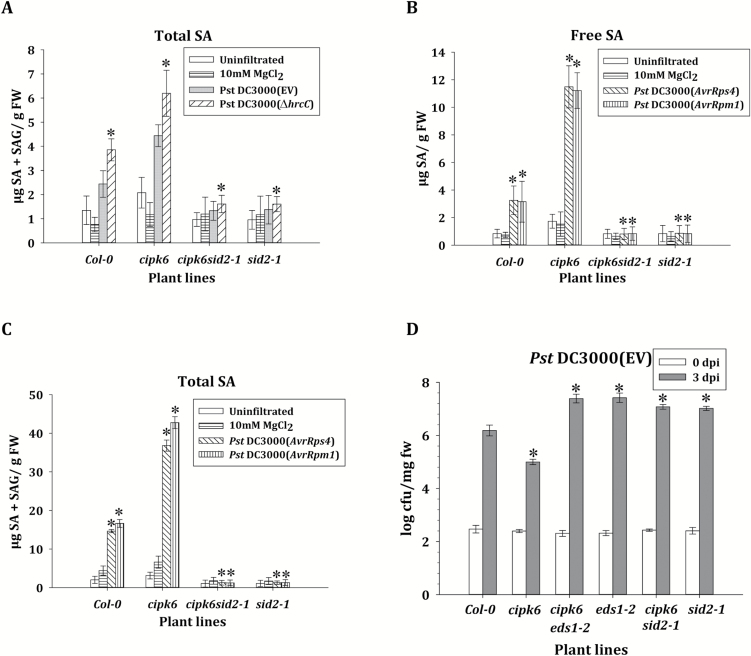
Negative regulation of pathogen resistance by CIPK6 was dependent on salicylic acid (SA). (A) Col-0, *cipk6*, *cipk6sid2-1*, and *sid2-1* leaves were manually infiltrated with MgCl_2_ and *Pst* DC3000 (EV) or *Pst* DC3000 (Δ*hrcC*). Total SA was measured at 9 hpi. (B, C) The same plant lines as above were infiltrated with MgCl_2_ and *Pst* DC3000 (*AvrRps4*/*AvrRpm1*). Total and free SA was measured at 9 hpi. (D) Bacterial growth was assessed at 3 dpi in Col-0, *cipk6*, *eds1-2*, *cipk6eds1-2*, *sid2-1*, and *cipk6sid2-1* plants and was expressed as the log cfu/mg fresh weight. The asterisks indicate a significant difference following two-way ANOVA (α=0.05).

SA accumulation upon pathogen infection is a hallmark of the defense response in plants against biotrophic and hemibiotrophic pathogens. SA is also a key regulator of plant immunity. Therefore, the expression level of *ICS1*, an important SA biosynthetic gene, was monitored in all four plant lines mentioned above. *ICS1* expression in response to *Pst* DC3000 was >3-fold higher in the *cipk6* line and 2-fold lower in *CIPK6OX* plants than that in wild-type and *CIPK6*-complemented *cipk6* plants from 2 dpi to 3 dpi (Fig. 3A). A similar difference in *ICS1* expression was observed in response to *Pst* DC3000-*AvrRps4*, -*AvrB*, and -*AvrRpm1* infection (Fig. 3B–D). Lack of expression or high expression of *CIPK6* did not affect *ICS1* expression in the case of *Pst* DC3000-*AvrRpt2* infection, similar to *PR1* expression (Figs 2E, 3E). SA undergoes various modifications to keep the balance between free active SA and total SA (Dempsey *et al.*, 2011). Since CIPK6 was found to modulate *ICS1* expression, total SA content was assayed in wild-type and *cipk6* plants after pathogen infiltration. *Pst* DC3000 infiltration in the *cipk6* line resulted in an ~2-fold higher accumulation of total SA than that in the wild-type (Col-0) plants (Fig. 4A). Infiltration with a *Pst* DC3000 strain with a disrupted type III secretion system, *Pst* DC3000 (Δ*hrcC*), resulted in accumulation of a much higher and differential level of SA in both the Col-0 and *cipk6* plants, most probably because the mutated pathogen is incapable of secreting PTI-suppressing type III effectors. To investigate the role of ICS1 in enhanced SA accumulation in *cipk6* plants, the total SA content was assayed in the *cipk6sid2-1* double mutant line after pathogen infiltration. The double mutant did not show any increase in total SA content upon infiltration, indicating that enhanced SA accumulation in *cipk6* plants was totally dependent on ICS1 (Fig. 4A). Similar enhanced and compromised accumulation of free and total SA was observed in *cipk6* and *cipk6sid2-1*, respectively, when infected with *Pst* DC3000 carrying *AvrRps4* or *AvrRpm1* (Fig. 4B, C). Compromise in SA accumulation upon *Pst* DC3000 infiltration by combined loss of function of both *CIPK6* and *ICS1* (*sid2-1*) was also reflected in disease susceptibility. The double mutant *cipk6sid2-1* exhibited an ~100-fold greater bacterial titer than *cipk6* plants, completely abolishing the resistance observed in the absence of CIPK6 (Fig. 4D). Induction of *PR1* expression was subsequently not observed in infected *cipk6sid2-1* plants at 48 hpi (Supplementary Fig. S3), showing that CIPK6-regulated defense modulation was dependent on SA accumulation. Similar higher bacterial growth and absence of induction of *PR1* expression were observed in *Pst* DC3000-infected *cipk6eds1-2*, suggesting that CIPK6 functions genetically upstream of EDS1 (Fig. 4D). External application of SA resulted in equivalent *PR1* gene expression in Col-0 and *cipk6* plants, indicating that CIPK6 does not function downstream of SA in Arabidopsis (Supplementary Fig. S4).

### CIPK6 negatively regulates ROS generation

**Fig. 5.**
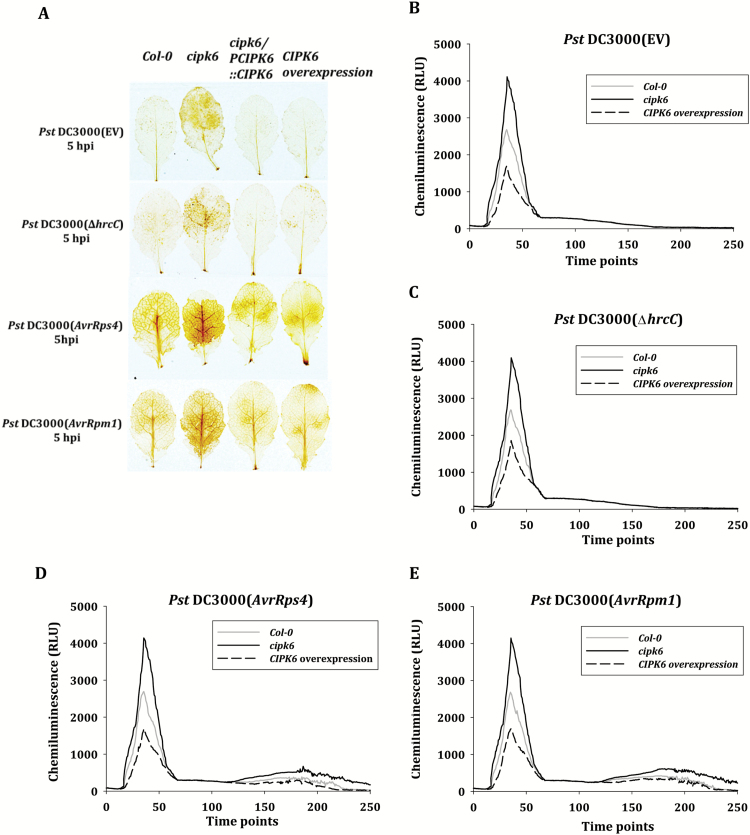
CIPK6 is a negative regulator of PTI- and ETI-triggered ROS generation. (A) Leaves of the Arabidopsis lines mentioned were manually infiltrated with *Pst* DC3000 (EV) (OD_600_=0.001), *Pst* DC3000 (Δ*hrcC*), *Pst* DC3000 (*AvrRps4*), or *Pst* DC3000 (*AvrRpm1*) (OD_600_=0.02). Hydrogen peroxide accumulation was detected by 3',3'-diaminobenzidine (DAB) staining at 5 hpi. (B–E) Time-course of ROS production in response to *Pst* DC3000 (EV), *Pst* DC3000 (Δ*hrcC*), *Pst* DC3000 (*AvrRps4*), and *Pst* DC3000 (*AvrRpm1*). ROS were measured for 250 min. The values presented are the mean of at least six biological replicates, each with four technical replicates. (This figure is available in colour at *JXB* online.)

Production of ROS is a hallmark of early PTI and ETI responses, and calcium sensors are known to be involved in the generation of elicitor-induced ROS, critical for the onset of the defense mechanism and the HR (Harding *et al.*, 1997). Hydrogen peroxide production was detected *in situ* using DAB in the leaves of Col-0, *cipk6*, *cipk6* /*PCIPK6::CIPK6* and *CIPK6OX* at 5 hpi with *Pst* DC3000 without and with *AvrRps4*/*AvrRpm1*. A relatively intense DAB stain showing production of hydrogen peroxide was observed in *cipk6* as compared with that in Col-0, *cipk6*/*PCIPK6::CIPK6*, and *CIPK6OX* plants in the case of all the bacterial strains including *Pst* DC3000 (Δ*hrcC*) (Fig. 5A). DAB stain in the case of *Pst* DC3000 with *AvrRps4* or *AvrRpm1* was more intense as these effectors elicit an effector-triggered immune response in Arabidopsis. The time course of ROS generation was analyzed by *in vivo* luminol-based assay to determine the involvement of CIPK6 in PTI- and ETI-mediated ROS generation against the same bacterial strains. *Pst* DC3000 and *Pst* DC3000 (Δ*hrcC*) generated only one ROS peak corresponding to PTI at 35 min post-inoculation while *Pst* DC3000 (*AvrRps4/ AvrRpm1*) generated two ROS peaks at 35 min and 180 min post-inoculation, denoting PTI and ETI, respectively (Fig. 5B–E). All the ROS peaks were substantially increased in *cipk6* plants as compared with Col-0 plants for all the pathogens, while ROS peaks were reduced to a much lower level in *CIPK6OX* plants. Further, the ETI-associated ROS peaks in *Pst* DC3000 (*AvrRps4/AvrRpm1*)-infected plants were prolonged in *cipk6* plants. Collectively, these data suggested that CIPK6 functions as a negative regulator of ROS generation during both PTI and ETI.

### CIPK6 negatively regulates MAPK-mediated gene expression during PTI

**Fig. 6.**
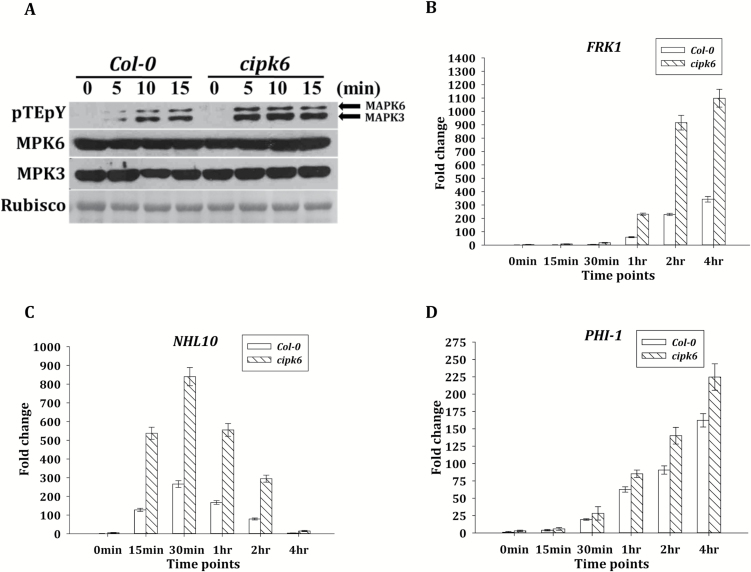
CIPK6 negatively regulated MAPK signaling during PTI. (A) Time course of MPK3 and MPK6 phosphorylation in Col-0 and *cipk6* plants upon flg22 treatment was assessed by western blot using antibody (pTEpY) specific for phosphorylated MAPKs. Total MPK3 and MPK6 proteins were detected by the respective protein-specific antibodies. Ponceau S staining of Rubisco was used as a loading control. (B–D) Expression analysis of *NHL10*, *FRK1*, and *PHI-1* in Col-0 and *cipk6* plants by qRT–PCR. The Col-0 and *cipk6* plants were treated with flg22 (1 µM). The samples were collected at the indicated time points for qRT–PCR analysis. *Actin 2* and *Tubulin 4* were used as internal controls. SDs were determined using three biological replicates and two technical replicates for each.

**Fig. 7.**
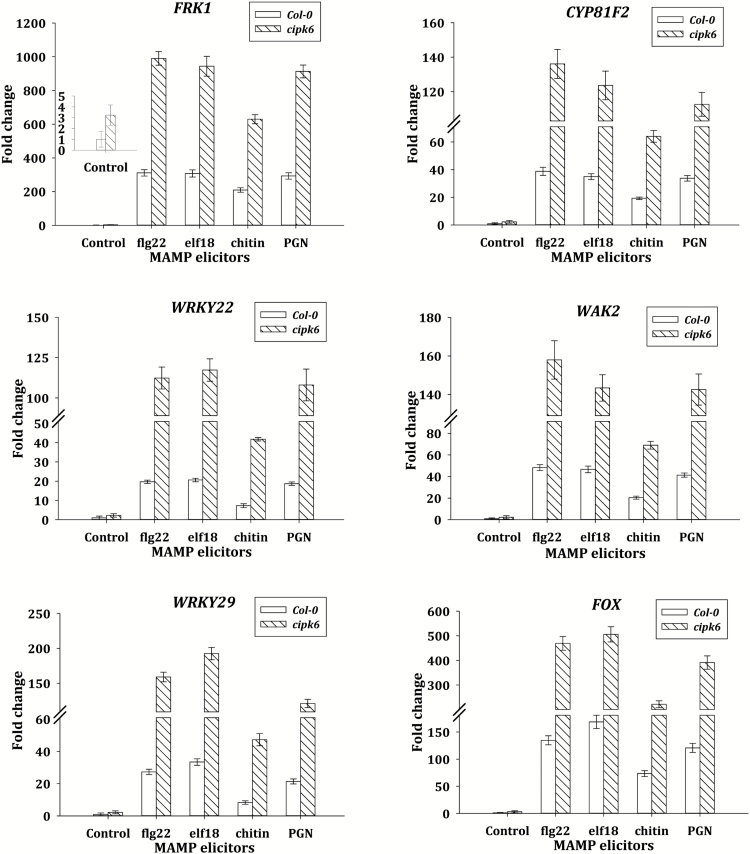
Expression analyses of PTI marker genes in response to various elicitors in the absence of CIPK6. qRT–PCR analysis of *FRK1*, *CYP81F2*, *WRKY22*, *WAK2*, *WRKY29*, and *FOX* expression in Col-0 and *cipk6* plants. Plants (8 d old, 21–23 °C) were treated with water, flg22 (1 µM), elf18 (1 µM), chitin (50 µg ml^–1^), or peptidoglycan (PGN; 50 µg ml^–1^). The samples were collected after 4 h of treatment for qRT–PCR analysis. The error bars in the qRT–PCR analysis indicate the SD. Expression of *Actin 2* and *Tubulin 4* was used as internal controls. At least three biological replicates for each sample were used for qRT–PCR analysis and at least two technical replicates were analyzed for each biological replicate.

As CIPK6 negatively regulated PTI-mediated ROS generation, its role in PTI-mediated gene expression was investigated using a well-known elicitor of PTI, flg22. Activation of the MAPK cascade and phosphorylation in the activation loops of MPK3 and MPK6 are the hallmarks of PTI signaling (Yung *et al.*, 1997; Asai *et al.*, 2002; Li *et al.*, 2007). While constitutive phosphorylation in the activation motifs of these MAPKs was not detectable in either of the plants, treatment with flg22 resulted in accelerated and enhanced phosphorylation of MPK3 and MPK6 in the *cipk6* plants in comparison with the wild-type plants (Fig. 6A). The role of CIPK6 in the PTI-mediated transcriptional activation was investigated by analyzing the expression patterns of early flg22-responsive genes such as *FRK1* (*FLG22-induced Receptor Kinase1*), *PHI-1* (*Phosphate Induced 1*), and *NHL10* (*NDR1/Hin1-Like 10*). Calcium-dependent protein kinase (CDPK) and MAPK cascade act differentially and synergistically to modulate transcriptional reprogramming. It was shown that flg22-induced expression of FRK1 was MAPK dependent (Asai *et al.*, 2002), whereas that of PHI-1 was CDPK dependent (Boudsocq *et al.*, 2010). NHL10 expression was shown to be equally activated by CDPK and MAPK cascades (Boudsocq *et al.*, 2010). flg22 elicited >4-fold higher expression of *FRK1* and *NHL10* in *cipk6* plants as compared with the wild-type plants, whereas no significant increase (<1.5-fold) was observed in *PHI-1* expression (Fig. 6B–D). Expression patterns of MAPK-dependent PTI marker genes, *FRK1*, *CYP81F2*, *WAK2*, *FOX*, *WRKY22*, and *WRKY29* (Boudsocq *et al.*, 2010), in response to flg22 and various bacterial and fungal elicitors, flg22, elf18, PGN, and chitin (Kunze *et al*, 2004; Sharp RG, 2013; Ao *et al.*, 2014), were analyzed. Expression levels of *CYP81F2*, *WAK2*, *FOX*, and *FRK1* were >3-fold higher, and those of *WRKY22* and *WRKY29* were >5-fold higher in *cipk6* plants as compared with their expression levels in Col-0 at 4 h post-treatment (Fig. 7). All these results suggested that CIPK6 negatively modulates the MAPK signaling pathway during PTI.

## Discussion

Calcium is a universal second messenger involved in modulation of diverse developmental and adaptive process. A rapid and sustained increase in the cytosolic calcium level ([Ca^2+^]_cyt_) is necessary for pathogen response (Grant *et al.*, 2000; Lecourieux *et al.*, 2006). An increase in free [Ca^2+^]_cyt_ is recognized by an array of Ca^2+^- sensors. Calmodulin (CaM) and calmodulin-like proteins (CMLs), a major group of Ca^2+^- sensors, are some of the key regulatory proteins of the plant immune response. CaM proteins were demonstrated to be transcriptional regulators and interactors of several plant immunity-associated proteins (Galon *et al.*, 2010). Direct regulation of the SA level by a Ca^2+^-binding protein has been demonstrated using AtSR1/CAMTA3, which negatively regulated the pathogen-induced SA level (Du *et al.*, 2009). Arabidopsis CML43 functions as an SA-inducible root-specific Ca^2+^- sensor (Bender *et al.*, 2014). The role of another group of calcium sensors, the CDPKs, particularly CPK4, -5, -6, and -11, in early signaling during PTI has been well demonstrated (Boudsocq *et al.*, 2010). Another group of Ca^2+^- sensors, CBLs, have been shown to play key roles in calcium-dependent processes in plants (Sanyal *et al.*, 2015). Recently, the Arabidopsis CBL-interacting protein kinase CIPK26 was shown to phosphorylate RbohF *in vitro*, and co-expression of AtCBL1 or AtCBL9 with AtCIPK26 was shown to enhance ROS production by RbohF in a human cell line (Drerup *et al.*, 2013). In tomato, the calcium sensor CBL10 and its interacting partner CIPK6 were shown to form a signaling module for ROS production and Pto-mediated resistance against *P. syringae* (de la Torre *et al.*, 2013). OsCIPK14/15 were shown to regulate microbe-associated hypersensitive cell death in rice cell culture (Kurusu *et al.*, 2010). In this study, we have shown that CIPK6, a component of the Ca^2+^ signaling pathway, functions as a negative regulator of the PAMP-triggered- and SA-mediated effector-triggered immune response in Arabidopsis. The CBL–CIPK signaling module functions as a Ca^2+^ decoding system (Batistič *et al.*, 2010), and phosphorylation of CBL by the interacting CIPK is necessary for full activity of this complex (Hashimoto *et al.*, 2012). Arabidopsis CIPK6 was shown to interact with CBL1, -2, -3, -4, and -9 in a yeast two-hybrid system and in plant cells (Lee *et al.*, 2007; Held *et al.*, 2011). Among these, AtCBL1 and -9 were shown to enhance RbohF phosphorylation by AtCIPK26 in a human cell line. The role of these CBL proteins in the plant immune response needs to be investigated.

Tomato and Arabidopsis show contrasting responses to AvrPto. While AvrPto triggers Pto-mediated defense in resistant tomato varieties, it suppresses PTI and enhances bacterial virulence by suppressing cell wall-based extracellular defense in Arabidopsis (Hauck *et al.*, 2003; Xiang *et al.*, 2008). Pathogen-induced expression patterns of *CIPK6* genes differ in these two species. *In silico* comparison of their upstream regulatory sequence showed very good similarity with respect to the presence of pathogen-responsive *cis*-acting elements, such as the WRKY-binding element (GTCAACG/TTCAACG) at ~1.0 kb upstream, the EIN3/EIL-binding element (ATGCA/ATGTA) at ~0.95 kb upstream, and the ERF-binding elements (TAGCT/TAGAG/TAGAA) at several positions upstream of the transcription start sites of both genes. EIN3 and ERF transcription factors are expressed in response to ethylene (Chao *et al.*, 1997). Tomato ERF proteins Pit4, Pit5, and Pit6 were shown to interact with Pto kinase and are involved in pathogen response (Zhou *et al.*, 1997). In contrast, Arabidopsis does not show AvrPto/Pto kinase-mediated ETI. This might be the reason for contrasting expression patterns of *CIPK6* genes in these two plant systems. However, our result demonstrated that CIPK6 negatively regulated defense against the bacterial effectors AvrRps4, AvrRpm1, and AvrB, which elicit ETI in Arabidopsis. Therefore, it appears that CIPK6 proteins perform distinct roles in different systems.

Unlike AvrRpm1 and AvrB, CIPK6 did not modulate resistance against AvrRpt2, although all of them target the common protein RIN4. However, AvrRpt2 is distinct from AvrRpm1 and AvrB as AvrRpt2 is a cysteine protease and functions by cleaving RIN4 (Chisholm *et al.*, 2005; Kim *et al.*, 2005), whereas the other two effectors induce phosphorylation of RIN4 (Mackey *et al.*, 2002). Therefore, the role of CIPK6 in phosphorylation of RIN4 needs further investigation. CIPK6 appears to act genetically upstream of EDS1 and, therefore, probably functions during recognition of the effectors or during PTI, as suggested by negative regulation of PTI-associated ROS production. Enhanced activation of the MAPK cascade and subsequent MAPK-dependent gene expression suggested that CIPK6 functions during PTI, probably upstream of MAPK activation, although the role of CIPK6 in multiple steps cannot be ruled out. Ca^2+^ has been recognized as the primary mediator of plant defense. CDPK and MAPK cascades were shown to act differentially as well as synergistically in this regulatory program (Boudsocq *et al.*, 2010). The absence of CIPK6 did not significantly alter the expression level of *PHI-1*, which is primarily regulated by CDPK after flg22 treatment. Further experiments are required in order to be able to comment on whether or not CIPK6 and CDPKs operate in different pathways.

Recently, Gutiérrez-Beltrán *et al.* (2017) have shown that SlCIPK6 interacts with and phosphorylates a universal stress protein, SlRd2. Co-expression of SlCIPK6 and SlRd2 in *Nicotiana benthamiana* resulted in reduced ROS generation. SlRd2 and Arabidopsis protein AtPHOS32 belong to the UspA protein family. AtPHOS32 was shown to be phosphorylated by MAPK3 and MAPK6 in response to flg22 treatment (Merkouropoulos *et al.*, 2008). We have shown that CIPK6 negatively regulated activation of the MAPK cascade. Hence, a co-ordinated role for MAPKs, and CIPK6 in regulating ROS generation during early PTI signaling needs to be investigated.

Plant immunity is a well-balanced process of positive and negative regulation of the immune response. In the absence of negative regulators, constitutive activation or overactivation of the defense response after infection would cause retarded plant growth or, in extreme conditions, even death of the plant (Heil and Baldwin, 2002; Nimchuk *et al.*, 2003). Previously, CaM and CaM-binding transcription factors were shown to regulate the plant immune response negatively (Kim *et al.*, 2002; Du *et al.*, 2009). Here, we show that a member of another major class of Ca^2+^-regulated proteins functions as a negative regulator of defense signaling in Arabidopsis.

Previously, we and others demonstrated that CIPK6 in Arabidopsis and *Brassica napus* functions as a positive regulator of abscisic acid (ABA) signaling and salinity tolerance (Tripathi *et al.*, 2009; Chen *et al.*, 2012; Tsou *et al.*, 2012). CIPK6 was shown to modulate the activity and plasma membrane targeting of the potassium transporter AKT2 (Held *et al.*, 2011). Dual and contrasting roles for AtCIPK6 in abiotic and biotic stress signaling illustrated its importance as a crosstalking node of two important plant signaling pathways.

## Supplementary data

Supplementary data are available at *JXB* online.

Fig. S1. Chlorosis in *Pst* DC3000-infected Arabidopsis lines.

Fig. S2. Time course of *CIPK6* expression after infection with *Pst* DC3000.

Fig. S3. *PR1* expression analysis in Col-0, *cipk6*, *cipk6eds1-2*, *eds1-2*, *cipk6sid2-1*, and *sid2-1* plants by qRT––PCR.

Fig. S4. The expression levels of *PR1* in Col-0 and *cipk6* in response to exogenous SA treatment were similar.

Table S1. List of primers used in the current study

## Supplementary Material

Supplementary Figures S1-S4 and Table S1
