# Supplementary material for: CBL-interacting protein kinase 6 negatively regulates immune response to Pseudomonas syringae in Arabidopsis
Source: J Exp Bot. 2017 May 24;68(13):3573–84. doi: 10.1093/jxb/erx170 (PMC5853215; doi:10.1093/jxb/erx170)
Supplement: Supplementary Figures S1-S4 and Table S1 [file erx170_suppl_supplementary_figures_s1_s4_table_s1.pdf]

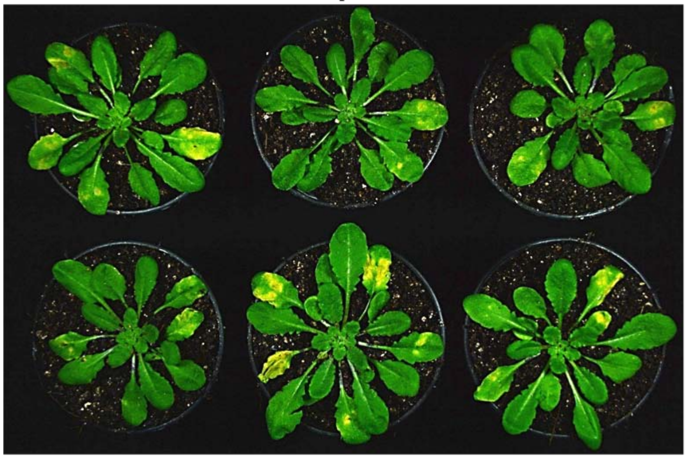

Figure S1. Chlorosis in Pst DC3000 infected *Arabidopsis* lines. *Pst* DC3000 (Empty Vector, EV) (OD600 = 0.0005) was manually infiltrated into leaves of various *Arabidopsis* plants mentioned. The infected leaves show the chlorosis at 3 dpi in the representative plants.

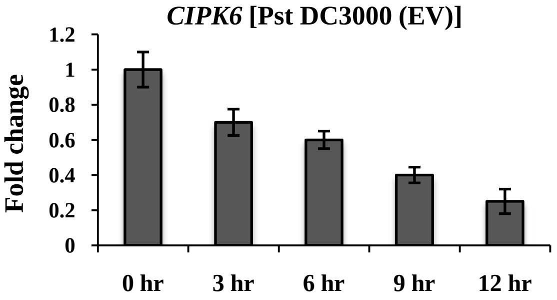

Figure S2. Time course of *CIPK6* expression after infection with *Pst* DC3000. Leaves of *Arabidopsis* (*Col-0*) plants were manually infiltrated with *Pst* DC3000 (EV) as described before and samples were harvested at the indicated time points for qRT-PCR analysis. *Actin 2* was used as internal control.

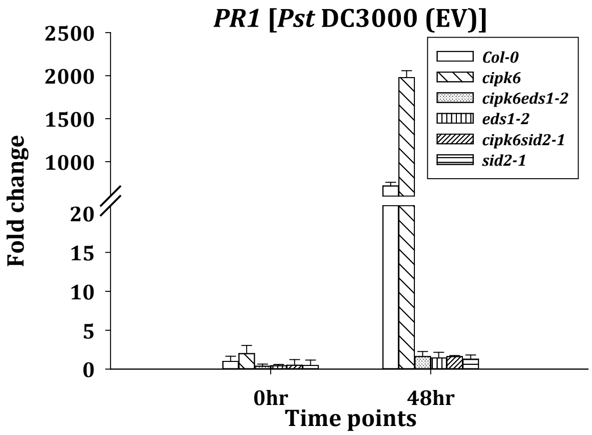

Figure S3. *PR1* expression analysis in *Col-0*, *cipk6*, *cipk6eds1-2*, *eds1-2*, *cipk6sid2-1* and *sid2-1* plants by qRT-PCR. *Actin 2* and *Tubulin 4* were used as internal control.

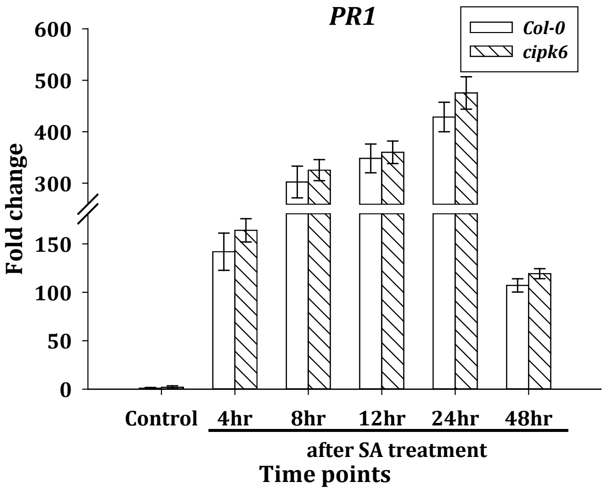

Figure S4. The expression levels of *PR1* in *Col-0* and *cipk6* in response to exogenous SA treatment were similar. The *Col-0* and *cipk6* leaves were sprayed with SA (0.5 mM) and harvested after indicated time period. Expression of *Actin 2* was used as internal control in qRT-PCR analysis.

Table S1. List of primers used in the current study.

S.N.	Name of primer	Sequence of primers
1.	GKBP_RP	5’-ATCAGATTGTCGTTTCCCGC-3’
2.	LBb1.3	5'-ATTTTGCCGATTTCGGAAC-3'
3.	CIPK6SpeI_F	5'-ACTAGTATGGTCGGAGCAAAACCGGTG-3'
4.	CIPK6SpeI_R	5'-ACTAGTTCAAGCAGGTGTAGAGGTCC-3'
5.	ProCIPK6_F	5'-GGCATGCCCTTGTTAACTTTTCAATGCTAAAG-3'
6.	CIPK6RNAiS_F	5'-CCATGGGGAAAGGGAAATTGGCGGTGGA-3'
7.	CIPK6RNAiS_R	5'-CTCGAGAACATATCGGCCACGACTC-3'
8.	CIPK6RNAiAS_F	5'-AAGCTTAACATATCGGCCACGACTC-3'
9.	CIPK6RNAiAS_R	5'-ACTAGTGGAAAGGGAAATTGGCGGTGGA-3'
10.	BjInt_F	5'-CTCGAGCCATGCTGCTCGTGGCAACA-3'
11.	BjInt_R	5'-AAGCTTGGTGTCTCGCTATGACCGA-3'
12.	ACT2 F	5'-GCCATCCAAGCTGTTCTCTC-3'
13.	ACT2 R	5'-GCTCGTAGTCAACAGCAACA-3'
14.	TUB4 RT_F	5'-AGGGAAACGAAGACAGCAAG-3'
15.	TUB4 RT_R	5'-GCTCGCTAATCCTACCTTTGG-3'
16.	ACT2 RT_F	5'-TCAGATGCCCAGAAGTCTTGTTC-3'
17.	ACT2 RT_R	5'-GTGGATTCCAGCAGCTTCCA-3'
18.	RealCIPK6_F	5'-AAGAAACAAGCAACGAGATCAAGA-3'
19.	RealCIPK6_R	5'-CACAATCACAACCACGGAAGA-3'
20.	PR1_RT_F	5'-AAGTGAGGTGTAACAATGGTGGAA-3'
21.	PR1_RT_R	5'-ACGTGTGTATGCATGATCACATCA-3'
22.	ICS1realF	5'-GGCTAATCTCCGCCGTCTCT-3'
23.	ICS1realR	5'-GACGCCGGAGGAAAACG-3'
24.	NHL10 RT_F	5'-TTCCTGTCCGTAACCCAAAC-3'
25.	NHL10 RT_R	5'-CCCTCGTAGTAGGCATGAGC-3'
26.	PHI1RT_F	5'-TTGGTTTAGACGGGATGGTG-3'
27.	PHI1RT_R	5'-ACTCCAGTACAAGCCGATCC-3'
28.	FRK1RT_F	5'-CGGTCAGATTTCAACAGTTGTC-3'
29.	FRK1RT_R	5'-AATAGCAGGTTGGCCTGTAATC-3'
30.	CYP81F2Real_F	5'-AAATGGAGAGAGCAACACAATG-3'
31.	CYP81F2Real_R	5'-ATCGCCCATTCCAATGTTAC-3'
32.	WAK2Real_F	5'-CGTGTGAGTACACAAATCATCG-3'
33.	WAK2Real_R	5'-TGGTTTAACCTCCTTTGTCTTC-3'
34.	FOXReal_F	5'-GGCTGCACTTCAACCCTTAC-3'
35.	FOXReal_R	5'-TTACTCTCTGTGGCGTTTGG-3'
36.	WRKY22_F	5'-TCCTTCGGAGAGATTCGAGA-3'
37.	WRKY22_R	5'-CTGCTGCTACATGGCACACT-3'
38.	WRKY29_F	5'-CCCGGAGAAATTCACCATAA-3'
39.	WRKY29_R	5'-ATCAGCGGATGGGATCATAG-3'
